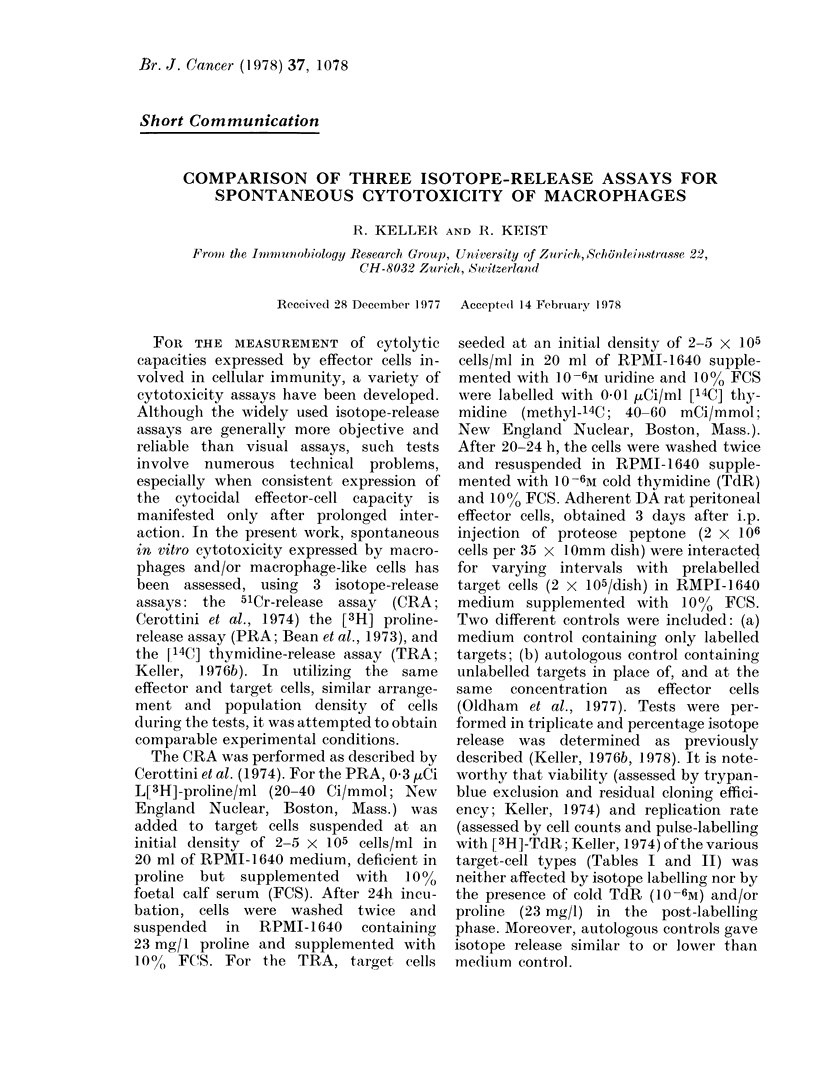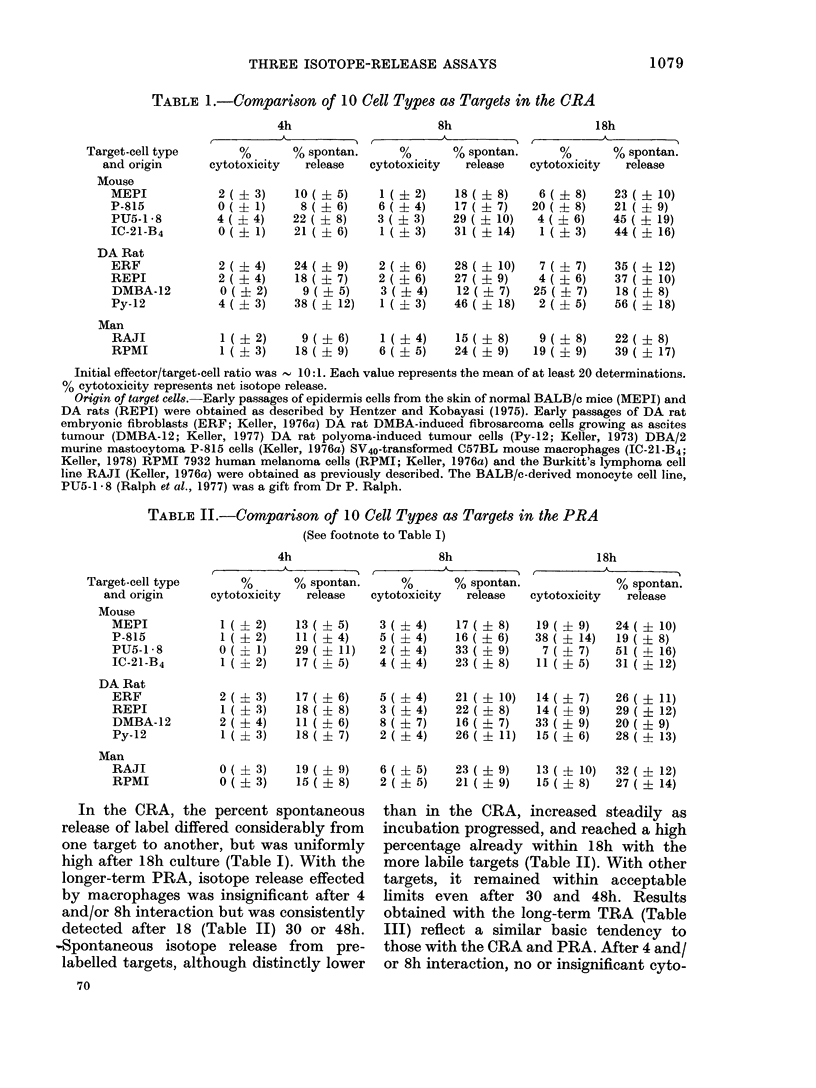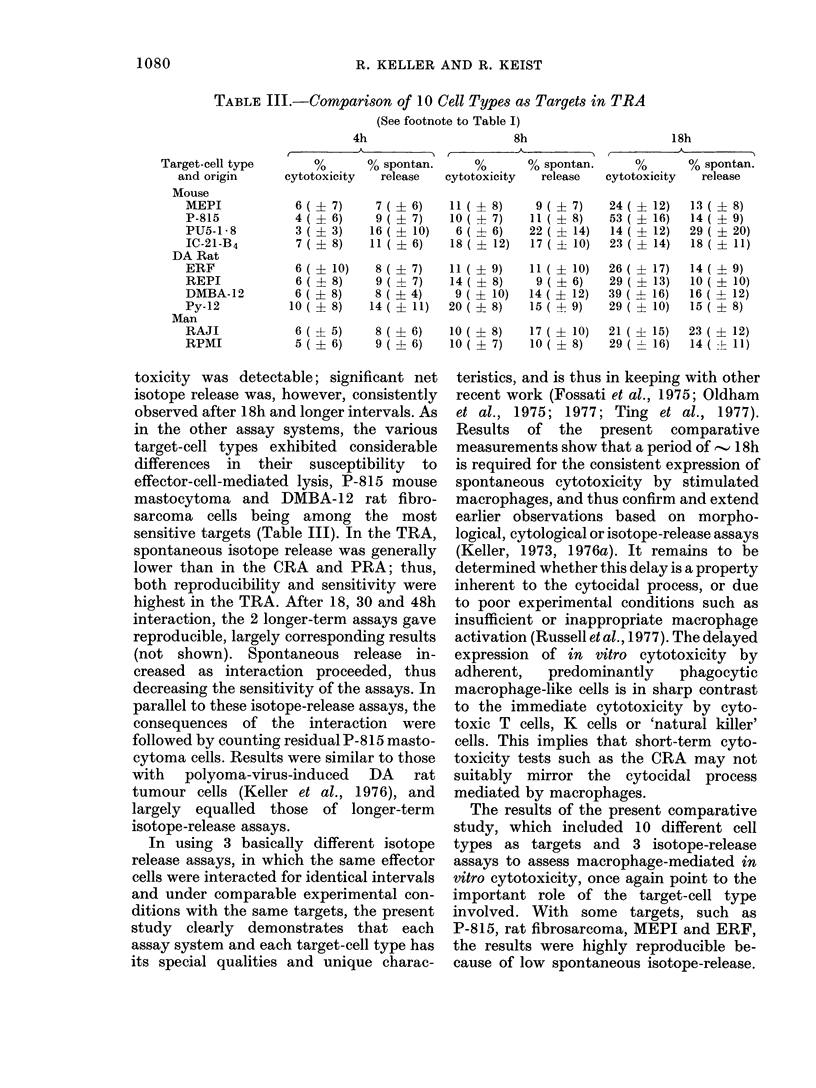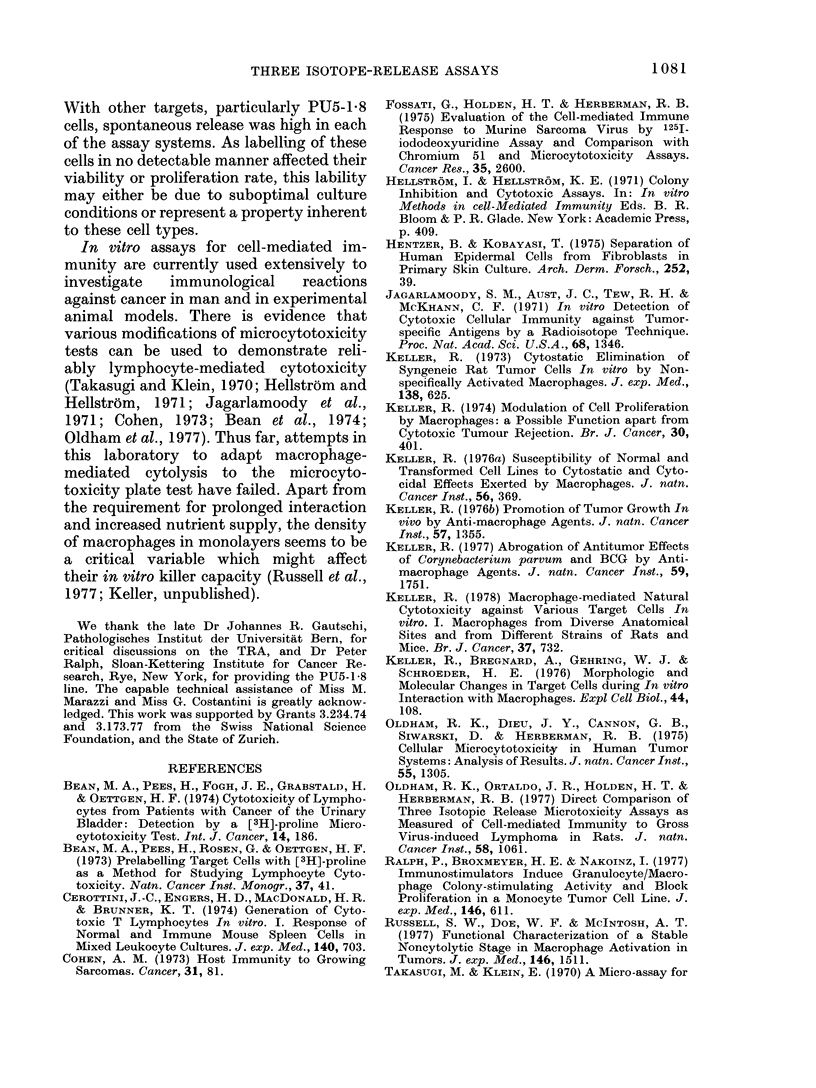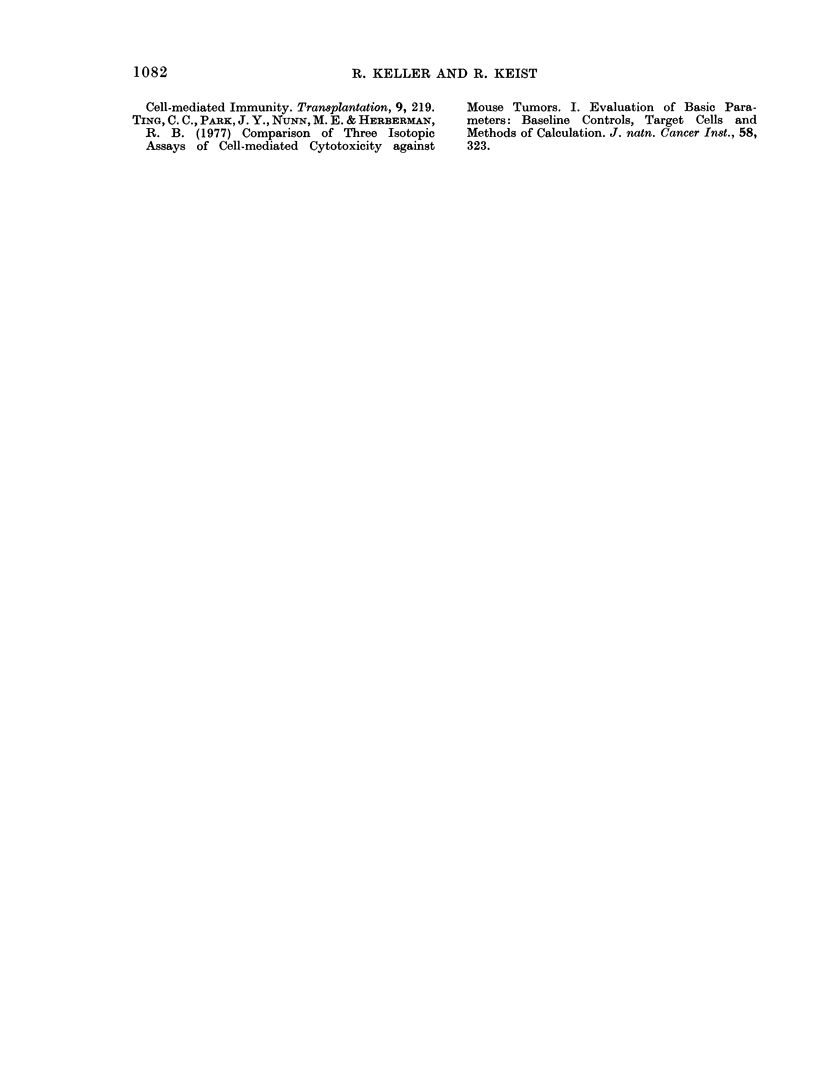# Comparison of three isotope-release assays for spontaneous cytotoxicity of macrophages.

**DOI:** 10.1038/bjc.1978.156

**Published:** 1978-06

**Authors:** R. Keller, R. Keist


*Br. J. Cancer* (1978) **37**, 1078

***Short Communication***

### COMPARISON OF THREE ISOTOPE-RELEASE ASSAYS FOR SPONTANEOUS CYTOTOXICITY OF MACROPHAGES

R. KELLER AND R. KEIST

*From the Immunobiology Research Group, University of Zurich, Schönleinstrasse 22, CH-8032 Zurich, Switzerland*

Received 28 December 1977 Accepted 14 February 1978

FOR THE MEASUREMENT of cytolytic capacities expressed by effector cells involved in cellular immunity, a variety of cytotoxicity assays have been developed. Although the widely used isotope-release assays are generally more objective and reliable than visual assays, such tests involve numerous technical problems, especially when consistent expression of the cytocidal effector-cell capacity is manifested only after prolonged interaction. In the present work, spontaneous *in vitro* cytotoxicity expressed by macrophages and/or macrophage-like cells has been assessed, using 3 isotope-release assays: the $^{51}$Cr-release assay (CRA; Cerottini *et al.*, 1974) the [$^{3}$H] proline-release assay (PRA; Bean *et al.*, 1973), and the [$^{14}$C] thymidine-release assay (TRA; Keller, 1976*b*). In utilizing the same effector and target cells, similar arrangement and population density of cells during the tests, it was attempted to obtain comparable experimental conditions.

The CRA was performed as described by Cerottini *et al.* (1974). For the PRA, 0·3 $\mu$Ci L[$^{3}$H]-proline/ml (20–40 Ci/mmol; New England Nuclear, Boston, Mass.) was added to target cells suspended at an initial density of 2–5 $\times$ $10^5$ cells/ml in 20 ml of RPMI-1640 medium, deficient in proline but supplemented with 10% foetal calf serum (FCS). After 24h incubation, cells were washed twice and suspended in RPMI-1640 containing 23 mg/l proline and supplemented with 10% FCS. For the TRA, target cells seeded at an initial density of 2–5 $\times$ $10^5$ cells/ml in 20 ml of RPMI-1640 supplemented with $10^{-6}$M uridine and 10% FCS were labelled with 0·01 $\mu$Ci/ml [$^{14}$C] thymidine (methyl-$^{14}$C; 40–60 mCi/mmol; New England Nuclear, Boston, Mass.). After 20–24 h, the cells were washed twice and resuspended in RPMI-1640 supplemented with $10^{-6}$M cold thymidine (TdR) and 10% FCS. Adherent DA rat peritoneal effector cells, obtained 3 days after i.p. injection of proteose peptone (2 $\times$ $10^6$ cells per 35 $\times$ 10mm dish) were interacted for varying intervals with prelabelled target cells (2 $\times$ $10^5$/dish) in RMPI-1640 medium supplemented with 10% FCS. Two different controls were included: (a) medium control containing only labelled targets; (b) autologous control containing unlabelled targets in place of, and at the same concentration as effector cells (Oldham *et al.*, 1977). Tests were performed in triplicate and percentage isotope release was determined as previously described (Keller, 1976*b*, 1978). It is noteworthy that viability (assessed by trypan-blue exclusion and residual cloning efficiency; Keller, 1974) and replication rate (assessed by cell counts and pulse-labelling with [$^{3}$H]-TdR; Keller, 1974) of the various target-cell types (Tables I and II) was neither affected by isotope labelling nor by the presence of cold TdR ($10^{-6}$M) and/or proline (23 mg/l) in the post-labelling phase. Moreover, autologous controls gave isotope release similar to or lower than medium control.

TABLE I.—*Comparison of* 10 *Cell Types as Targets in the CRA*

| Target-cell type and origin | 4h % cytotoxicity | 4h % spontan. release | 8h % cytotoxicity | 8h % spontan. release | 18h % cytotoxicity | 18h % spontan. release |
|---|---|---|---|---|---|---|
| Mouse | | | | | | |
| MEPI | 2 ( ± 3) | 10 ( ± 5) | 1 ( ± 2) | 18 ( ± 8) | 6 ( ± 8) | 23 ( ± 10) |
| P-815 | 0 ( ± 1) | 8 ( ± 6) | 6 ( ± 4) | 17 ( ± 7) | 20 ( ± 8) | 21 ( ± 9) |
| PU5-1·8 | 4 ( ± 4) | 22 ( ± 8) | 3 ( ± 3) | 29 ( ± 10) | 4 ( ± 6) | 45 ( ± 19) |
| IC-21-$B_4$ | 0 ( ± 1) | 21 ( ± 6) | 1 ( ± 3) | 31 ( ± 14) | 1 ( ± 3) | 44 ( ± 16) |
| DA Rat | | | | | | |
| ERF | 2 ( ± 4) | 24 ( ± 9) | 2 ( ± 6) | 28 ( ± 10) | 7 ( ± 7) | 35 ( ± 12) |
| REPI | 2 ( ± 4) | 18 ( ± 7) | 2 ( ± 6) | 27 ( ± 9) | 4 ( ± 6) | 37 ( ± 10) |
| DMBA-12 | 0 ( ± 2) | 9 ( ± 5) | 3 ( ± 4) | 12 ( ± 7) | 25 ( ± 7) | 18 ( ± 8) |
| Py-12 | 4 ( ± 3) | 38 ( ± 12) | 1 ( ± 3) | 46 ( ± 18) | 2 ( ± 5) | 56 ( ± 18) |
| Man | | | | | | |
| RAJI | 1 ( ± 2) | 9 ( ± 6) | 1 ( ± 4) | 15 ( ± 8) | 9 ( ± 8) | 22 ( ± 8) |
| RPMI | 1 ( ± 3) | 18 ( ± 9) | 6 ( ± 5) | 24 ( ± 9) | 19 ( ± 9) | 39 ( ± 17) |

Initial effector/target-cell ratio was ~ 10:1. Each value represents the mean of at least 20 determinations. % cytotoxicity represents net isotope release.

*Origin of target cells.*—Early passages of epidermis cells from the skin of normal BALB/c mice (MEPI) and DA rats (REPI) were obtained as described by Hentzer and Kobayasi (1975). Early passages of DA rat embryonic fibroblasts (ERF; Keller, 1976*a*) DA rat DMBA-induced fibrosarcoma cells growing as ascites tumour (DMBA-12; Keller, 1977) DA rat polyoma-induced tumour cells (Py-12; Keller, 1973) DBA/2 murine mastocytoma P-815 cells (Keller, 1976*a*) $SV_{40}$-transformed C57BL mouse macrophages (IC-21-$B_4$; Keller, 1978) RPMI 7932 human melanoma cells (RPMI; Keller, 1976*a*) and the Burkitt's lymphoma cell line RAJI (Keller, 1976*a*) were obtained as previously described. The BALB/c-derived monocyte cell line, PU5-1·8 (Ralph *et al.*, 1977) was a gift from Dr P. Ralph.

TABLE II.—*Comparison of* 10 *Cell Types as Targets in the PRA*

(See footnote to Table I)

| Target-cell type and origin | 4h % cytotoxicity | 4h % spontan. release | 8h % cytotoxicity | 8h % spontan. release | 18h cytotoxicity | 18h % spontan. release |
|---|---|---|---|---|---|---|
| Mouse | | | | | | |
| MEPI | 1 ( ± 2) | 13 ( ± 5) | 3 ( ± 4) | 17 ( ± 8) | 19 ( ± 9) | 24 ( ± 10) |
| P-815 | 1 ( ± 2) | 11 ( ± 4) | 5 ( ± 4) | 16 ( ± 6) | 38 ( ± 14) | 19 ( ± 8) |
| PU5-1·8 | 0 ( ± 1) | 29 ( ± 11) | 2 ( ± 4) | 33 ( ± 9) | 7 ( ± 7) | 51 ( ± 16) |
| IC-21-$B_4$ | 1 ( ± 2) | 17 ( ± 5) | 4 ( ± 4) | 23 ( ± 8) | 11 ( ± 5) | 31 ( ± 12) |
| DA Rat | | | | | | |
| ERF | 2 ( ± 3) | 17 ( ± 6) | 5 ( ± 4) | 21 ( ± 10) | 14 ( ± 7) | 26 ( ± 11) |
| REPI | 1 ( ± 3) | 18 ( ± 8) | 3 ( ± 4) | 22 ( ± 8) | 14 ( ± 9) | 29 ( ± 12) |
| DMBA-12 | 2 ( ± 4) | 11 ( ± 6) | 8 ( ± 7) | 16 ( ± 7) | 33 ( ± 9) | 20 ( ± 9) |
| Py-12 | 1 ( ± 3) | 18 ( ± 7) | 2 ( ± 4) | 26 ( ± 11) | 15 ( ± 6) | 28 ( ± 13) |
| Man | | | | | | |
| RAJI | 0 ( ± 3) | 19 ( ± 9) | 6 ( ± 5) | 23 ( ± 9) | 13 ( ± 10) | 32 ( ± 12) |
| RPMI | 0 ( ± 3) | 15 ( ± 8) | 2 ( ± 5) | 21 ( ± 9) | 15 ( ± 8) | 27 ( ± 14) |

In the CRA, the percent spontaneous release of label differed considerably from one target to another, but was uniformly high after 18h culture (Table I). With the longer-term PRA, isotope release effected by macrophages was insignificant after 4 and/or 8h interaction but was consistently detected after 18 (Table II) 30 or 48h. Spontaneous isotope release from pre-labelled targets, although distinctly lower than in the CRA, increased steadily as incubation progressed, and reached a high percentage already within 18h with the more labile targets (Table II). With other targets, it remained within acceptable limits even after 30 and 48h. Results obtained with the long-term TRA (Table III) reflect a similar basic tendency to those with the CRA and PRA. After 4 and/ or 8h interaction, no or insignificant cyto-

TABLE III.—*Comparison of* 10 *Cell Types as Targets in TRA*

(See footnote to Table I)

| Target-cell type and origin | 4h % cytotoxicity | 4h % spontan. release | 8h % cytotoxicity | 8h % spontan. release | 18h % cytotoxicity | 18h % spontan. release |
|---|---|---|---|---|---|---|
| Mouse | | | | | | |
| MEPI | 6 (± 7) | 7 (± 6) | 11 (± 8) | 9 (± 7) | 24 (± 12) | 13 (± 8) |
| P-815 | 4 (± 6) | 9 (± 7) | 10 (± 7) | 11 (± 8) | 53 (± 16) | 14 (± 9) |
| PU5-1·8 | 3 (± 3) | 16 (± 10) | 6 (± 6) | 22 (± 14) | 14 (± 12) | 29 (± 20) |
| IC-21-$B_4$ | 7 (± 8) | 11 (± 6) | 18 (± 12) | 17 (± 10) | 23 (± 14) | 18 (± 11) |
| DA Rat | | | | | | |
| ERF | 6 (± 10) | 8 (± 7) | 11 (± 9) | 11 (± 10) | 26 (± 17) | 14 (± 9) |
| REPI | 6 (± 8) | 9 (± 7) | 14 (± 8) | 9 (± 6) | 29 (± 13) | 10 (± 10) |
| DMBA-12 | 6 (± 8) | 8 (± 4) | 9 (± 10) | 14 (± 12) | 39 (± 16) | 16 (± 12) |
| Py-12 | 10 (± 8) | 14 (± 11) | 20 (± 8) | 15 (± 9) | 29 (± 10) | 15 (± 8) |
| Man | | | | | | |
| RAJI | 6 (± 5) | 8 (± 6) | 10 (± 8) | 17 (± 10) | 21 (± 15) | 23 (± 12) |
| RPMI | 5 (± 6) | 9 (± 6) | 10 (± 7) | 10 (± 8) | 29 (± 16) | 14 (± 11) |

toxicity was detectable; significant net isotope release was, however, consistently observed after 18h and longer intervals. As in the other assay systems, the various target-cell types exhibited considerable differences in their susceptibility to effector-cell-mediated lysis, P-815 mouse mastocytoma and DMBA-12 rat fibrosarcoma cells being among the most sensitive targets (Table III). In the TRA, spontaneous isotope release was generally lower than in the CRA and PRA; thus, both reproducibility and sensitivity were highest in the TRA. After 18, 30 and 48h interaction, the 2 longer-term assays gave reproducible, largely corresponding results (not shown). Spontaneous release increased as interaction proceeded, thus decreasing the sensitivity of the assays. In parallel to these isotope-release assays, the consequences of the interaction were followed by counting residual P-815 mastocytoma cells. Results were similar to those with polyoma-virus-induced DA rat tumour cells (Keller *et al.*, 1976), and largely equalled those of longer-term isotope-release assays.

In using 3 basically different isotope release assays, in which the same effector cells were interacted for identical intervals and under comparable experimental conditions with the same targets, the present study clearly demonstrates that each assay system and each target-cell type has its special qualities and unique characteristics, and is thus in keeping with other recent work (Fossati *et al.*, 1975; Oldham *et al.*, 1975; 1977; Ting *et al.*, 1977). Results of the present comparative measurements show that a period of ~ 18h is required for the consistent expression of spontaneous cytotoxicity by stimulated macrophages, and thus confirm and extend earlier observations based on morphological, cytological or isotope-release assays (Keller, 1973, 1976*a*). It remains to be determined whether this delay is a property inherent to the cytocidal process, or due to poor experimental conditions such as insufficient or inappropriate macrophage activation (Russell *et al.*, 1977). The delayed expression of *in vitro* cytotoxicity by adherent, predominantly phagocytic macrophage-like cells is in sharp contrast to the immediate cytotoxicity by cytotoxic T cells, K cells or ‘natural killer’ cells. This implies that short-term cytotoxicity tests such as the CRA may not suitably mirror the cytocidal process mediated by macrophages.

The results of the present comparative study, which included 10 different cell types as targets and 3 isotope-release assays to assess macrophage-mediated *in vitro* cytotoxicity, once again point to the important role of the target-cell type involved. With some targets, such as P-815, rat fibrosarcoma, MEPI and ERF, the results were highly reproducible because of low spontaneous isotope-release.

With other targets, particularly PU5-1·8 cells, spontaneous release was high in each of the assay systems. As labelling of these cells in no detectable manner affected their viability or proliferation rate, this lability may either be due to suboptimal culture conditions or represent a property inherent to these cell types.

*In vitro* assays for cell-mediated immunity are currently used extensively to investigate immunological reactions against cancer in man and in experimental animal models. There is evidence that various modifications of microcytotoxicity tests can be used to demonstrate reliably lymphocyte-mediated cytotoxicity (Takasugi and Klein, 1970; Hellström and Hellström, 1971; Jagarlamoody *et al.*, 1971; Cohen, 1973; Bean *et al.*, 1974; Oldham *et al.*, 1977). Thus far, attempts in this laboratory to adapt macrophage-mediated cytolysis to the microcytotoxicity plate test have failed. Apart from the requirement for prolonged interaction and increased nutrient supply, the density of macrophages in monolayers seems to be a critical variable which might affect their *in vitro* killer capacity (Russell *et al.*, 1977; Keller, unpublished).

We thank the late Dr Johannes R. Gautschi, Pathologisches Institut der Universität Bern, for critical discussions on the TRA, and Dr Peter Ralph, Sloan-Kettering Institute for Cancer Research, Rye, New York, for providing the PU5-1·8 line. The capable technical assistance of Miss M. Marazzi and Miss G. Costantini is greatly acknowledged. This work was supported by Grants 3.234.74 and 3.173.77 from the Swiss National Science Foundation, and the State of Zurich.

### REFERENCES

BEAN, M. A., PEES, H., FOGH, J. E., GRABSTALD, H. & OETTGEN, H. F. (1974) Cytotoxicity of Lymphocytes from Patients with Cancer of the Urinary Bladder: Detection by a [$^{3}$H]-proline Microcytotoxicity Test. *Int. J. Cancer*, **14**, 186.

BEAN, M. A., PEES, H., ROSEN, G. & OETTGEN, H. F. (1973) Prelabelling Target Cells with [$^{3}$H]-proline as a Method for Studying Lymphocyte Cytotoxicity. *Natn. Cancer Inst. Monogr.*, **37**, 41.

CEROTTINI, J.-C., ENGERS, H. D., MACDONALD, H. R. & BRUNNER, K. T. (1974) Generation of Cytotoxic T Lymphocytes *In vitro*. I. Response of Normal and Immune Mouse Spleen Cells in Mixed Leukocyte Cultures. *J. exp. Med.*, **140**, 703.

COHEN, A. M. (1973) Host Immunity to Growing Sarcomas. *Cancer*, **31**, 81.

FOSSATI, G., HOLDEN, H. T. & HERBERMAN, R. B. (1975) Evaluation of the Cell-mediated Immune Response to Murine Sarcoma Virus by $^{125}$I-iododeoxyuridine Assay and Comparison with Chromium 51 and Microcytotoxicity Assays. *Cancer Res.*, **35**, 2600.

HELLSTRÖM, I. & HELLSTRÖM, K. E. (1971) Colony Inhibition and Cytotoxic Assays. In: *In vitro Methods in cell-Mediated Immunity* Eds. B. R. Bloom & P. R. Glade. New York: Academic Press, p. 409.

HENTZER, B. & KOBAYASI, T. (1975) Separation of Human Epidermal Cells from Fibroblasts in Primary Skin Culture. *Arch. Derm. Forsch.*, **252**, 39.

JAGARLAMOODY, S. M., AUST, J. C., TEW, R. H. & MCKHANN, C. F. (1971) *In vitro* Detection of Cytotoxic Cellular Immunity against Tumor-specific Antigens by a Radioisotope Technique. *Proc. Nat. Acad. Sci. U.S.A.*, **68**, 1346.

KELLER, R. (1973) Cytostatic Elimination of Syngeneic Rat Tumor Cells *In vitro* by Non-specifically Activated Macrophages. *J. exp. Med.*, **138**, 625.

KELLER, R. (1974) Modulation of Cell Proliferation by Macrophages: a Possible Function apart from Cytotoxic Tumour Rejection. *Br. J. Cancer*, **30**, 401.

KELLER, R. (1976*a*) Susceptibility of Normal and Transformed Cell Lines to Cytostatic and Cytocidal Effects Exerted by Macrophages. *J. natn. Cancer Inst.*, **56**, 369.

KELLER, R. (1976*b*) Promotion of Tumor Growth *In vivo* by Anti-macrophage Agents. *J. natn. Cancer Inst.*, **57**, 1355.

KELLER, R. (1977) Abrogation of Antitumor Effects of *Corynebacterium parvum* and BCG by Anti-macrophage Agents. *J. natn. Cancer Inst.*, **59**, 1751.

KELLER, R. (1978) Macrophage-mediated Natural Cytotoxicity against Various Target Cells *In vitro*. I. Macrophages from Diverse Anatomical Sites and from Different Strains of Rats and Mice. *Br. J. Cancer*, **37**, 732.

KELLER, R., BREGNARD, A., GEHRING, W. J. & SCHROEDER, H. E. (1976) Morphologic and Molecular Changes in Target Cells during *In vitro* Interaction with Macrophages. *Expl Cell Biol.*, **44**, 108.

OLDHAM, R. K., DIEU, J. Y., CANNON, G. B., SIWARSKI, D. & HERBERMAN, R. B. (1975) Cellular Microcytotoxicity in Human Tumor Systems: Analysis of Results. *J. natn. Cancer Inst.*, **55**, 1305.

OLDHAM, R. K., ORTALDO, J. R., HOLDEN, H. T. & HERBERMAN, R. B. (1977) Direct Comparison of Three Isotopic Release Microtoxicity Assays as Measured of Cell-mediated Immunity to Gross Virus-induced Lymphoma in Rats. *J. natn. Cancer Inst.*, **58**, 1061.

RALPH, P., BROXMEYER, H. E. & NAKOINZ, I. (1977) Immunostimulators Induce Granulocyte/Macrophage Colony-stimulating Activity and Block Proliferation in a Monocyte Tumor Cell Line. *J. exp. Med.*, **146**, 611.

RUSSELL, S. W., DOE, W. F. & MCINTOSH, A. T. (1977) Functional Characterization of a Stable Noncytolytic Stage in Macrophage Activation in Tumors. *J. exp. Med.*, **146**, 1511.

TAKASUGI, M. & KLEIN, E. (1970) A Micro-assay for

Cell-mediated Immunity. *Transplantation*, **9**, 219.
TING, C. C., PARK, J. Y., NUNN, M. E. & HERBERMAN, R. B. (1977) Comparison of Three Isotopic Assays of Cell-mediated Cytotoxicity against Mouse Tumors. I. Evaluation of Basic Parameters: Baseline Controls, Target Cells and Methods of Calculation. *J. natn. Cancer Inst.*, **58**, 323.